# Correlation between Cardiac Ultrasound Index and Cardiovascular Risk in Healthy Obese and Overweight Populations

**DOI:** 10.1155/2022/2235994

**Published:** 2022-09-21

**Authors:** Xiaoyu Pan, Xiaoyi Chen, Lin Ren, Zelin Li, Shuchun Chen

**Affiliations:** ^1^Department of Internal Medicine, Hebei Medical University, Shijiazhuang, Hebei, China; ^2^Department of Endocrinology, Hebei General Hospital, Shijiazhuang, Hebei, China

## Abstract

**Objective:**

To investigate the correlation of obesity and overweight with cardiac ultrasound parameters and future cardiovascular risk among healthy populations.

**Methods:**

Basic clinical characteristics as well as cardiac ultrasound parameters were collected from healthy people. Firstly, all participants were divided into three groups: normal, overweight, and obese. Then the differences in cardiac ultrasound parameters between the three groups were calculated. Subsequently, those aged 35–60 years were screened to determine their cardiovascular risk according to the SCORE system. Finally, the correlation between cardiac ultrasound indices and cardiovascular risk was calculated.

**Results:**

A total of 1328 healthy participants were included, of whom 504 were normal, 580 were overweight and 244 were obese. Obesity and overweight significantly increased the aorta, left atrium, right atrium, right ventricle, the end-diastolic diameter of the left ventricle, main pulmonary artery, right ventricular outflow tract, interventricular septum, left ventricular posterior wall, and triglycerides and decreased E/A values and high-density lipoprotein-cholesterol. Ejection fraction, fractional shortening, low-density lipoprotein-cholesterol, and total cholesterol did not change between the three groups. A total of 781 participants were screened for SCORE scores. Obesity and being overweight significantly increased the incidence of future cardiovascular events, and lower E/A values were also associated with cardiovascular risk. All cardiac parameters were strongly associated with cardiovascular risk.

**Conclusion:**

Our research demonstrates that obesity and overweight can damage heart shape and function and raise the risk of future cardiovascular events in people that are healthy. Cardiovascular risk and cardiac structural and functional impairments are significantly positively correlated.

## 1. Introduction

Global economic development is raising living standards, which has also led to changes in the disease spectrum in urban and rural areas, a decrease in infectious diseases, an increase in per capita life expectancy, and a sharp increase in the morbidity and mortality of cardiovascular diseases, particularly coronary heart disease, structural heart disease, and hypertension, compared to 30 years ago. The development of irreversible heart failure, which has a considerable negative impact on a patient's quality of life and greatly increases healthcare expenses, may result from structural heart function anomalies once certain cardiovascular diseases have advanced to a particular stage. Appropriate interventions can be taken to halt or slow the progression of cardiovascular disease when it is still in its early stages or when only minor structural alterations are present [1]. Cigarette smoking, dyslipidemia, hypertension, and metabolic syndrome are all risk factors for cardiovascular diseases [2]. The prevalence of obesity and overweight has considerably increased as a result of dietary changes and a lack of physical activity, which are the most unappreciated and major risk factors for cardiovascular disease. The association between obesity or being overweight and cardiovascular disease has been established by numerous studies [3–5]. The relationship between obesity or being overweight and cardiovascular diseases needs to be further studied.

The quick, noninvasive, and repeatable process of echocardiography can be used to assess the anatomy and function of the heart. Research has shown that many cardiovascular disorders that act as triggers already undergo adaptive changes in heart structure and function early in their development and that these changes frequently manifest long before the onset of clinical symptoms [6]. Therefore, routine cardiac ultrasonography allows for early detection of anomalies in the heart structure and function in those with high cardiovascular disease risk factors, allowing for appropriate therapy to slow the disease's course. The SCORE system, a commonly used method based on the study results, is one technique to assess cardiovascular risk. The SCORE score generates a numerically demarcated scale by evaluating a patient's chance of experiencing their first fatal cardiovascular event within the next 10 years based on age, sex, smoking, systolic blood pressure, and total cholesterol levels. Individual cardiovascular risk is categorized into four categories: extremely high (>10% chance of cardiovascular death within 10 years), high (5–10%), intermediate (1%–5%), and low (<1%) [7, 8]. At least every five years, those over 40 should have a risk assessment done.

However, there is scant evidence connecting cardiac morphological and functional indices to cardiovascular risk factors in healthy individuals. Therefore, from the standpoint of cardiac structure and function, the population's future risk of cardiovascular disease can be directly assessed, enabling targeted interventions. This can be achieved by looking at the relationship between cardiovascular risk factors and cardiac ultrasonography indicators. Given the significance of obesity as a risk factor for cardiovascular disease, the current study focused on examining (1) the association of obesity and overweight with cardiac structure and function in a healthy population, (2) the relevance of obesity and overweight to future risk of cardiovascular disease, and (3) the relationship between cardiovascular risk and cardiac structural and functional indices.

## 2. Materials and Methods

### 2.1. Study Population

It was an outpatient-based, cross-sectional study. It took place in Hebei General Hospital's Medical Examination Center. The protocol was approved by the Ethics Committee of Hebei General Hospital (the number of the ethics committee: No. 202027). Before enrollment, an informed consent form was signed by each population. The Declaration of Helsinki was followed when conducting the study.

The population included in this study was the healthy population who were examined at the physical examination center of the Hebei General Hospital from June 1, 2022, to June 18, 2022. Inclusion criteria: healthy population with no previous disease affecting the structure and function of the heart. Exclusion criteria: (1) diabetes mellitus; (2) hypertension; (3) coronary artery disease; (4) history of previous cardiac surgery; (5) cardiac insufficiency, hepatic or renal impairment; (6) pregnancy or lactation; (7) malignancy; and (8) other diseases or medications that affect cardiac function or structure.

### 2.2. Information and Data Collection

Basic information was collected from all participants, including gender, age, tobacco use, drug use, and blood pressure. The body mass index (BMI) was calculated based on height and weight. All participants had to complete cardiac ultrasound, including the aorta (AO), left atrium (LA), right atrium (RA), right ventricle (RV), end diastolic diameter of left ventricle (EDD), main pulmonary artery (MPA), right ventricular outflow tract (RVOT), ejection fraction (EF), fractional shortening (FS), interventricular septum (IVS), left ventricular posterior wall (LVPW), and E/A. Blood samples were collected from all participants in a state of fasting for more than 8 hours. Blood markers include high-density lipoprotein-cholesterol (HDL-C), low-density lipoprotein-cholesterol (LDL-C), triglycerides (TG), and total cholesterol (TC). All indicators are tested according to uniform standards.

### 2.3. Assessment of Cardiovascular Risk

The cardiovascular risk SCORE system is one of the most commonly used cardiovascular risk assessment methods. It assesses a patient's risk of having a first fatal cardiovascular event in the next 10 years based on five factors: age, sex, smoking, systolic blood pressure, and TC level. The higher the number of the above five factors combined, the higher the risk.

### 2.4. Statistical Analysis

Continuous data were expressed as the mean ± standard deviation (S.D.). One-way analysis of variance (ANOVA) was used to compare continuous variables between the three groups. Categorical variables were analyzed using chi-square tests. A Spearman or Pearson correlation analysis was used to analyze the correlation between the dependent and independent variables. The analysis of all the data was performed using GraphPad Prism 8.01 software. Statistical significance was considered when the two-tailed *P* value was <0.05.

## 3. Results

### 3.1. Baseline Characteristics of All Participants

A total of 1328 healthy participants were included (Supplemental Figure 1). We grouped all participants according to BMI, with normal defined as BMI < 24, overweight as 28 > BMI ≥ 24, and obese as BMI ≥ 28, with 504 normal, 580 overweight, and 244 obese. Details of age, gender, BMI, blood pressure, tobacco use, lipids, and cardiac ultrasound parameters for all three groups of participants are presented in Table 1.

### 3.2. Cardiac Ultrasound Parameters

**Figure 1:**
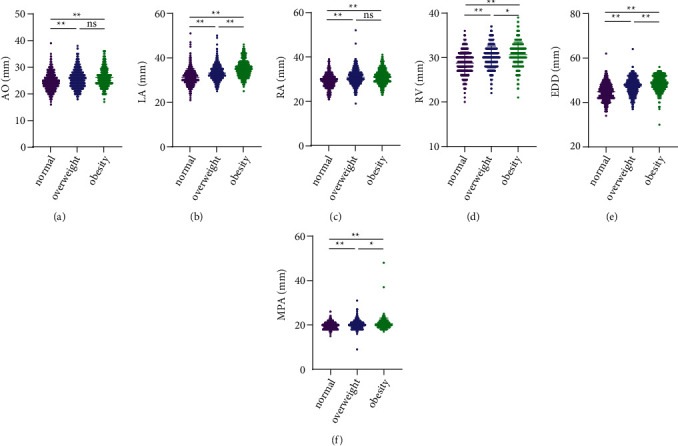
Comparison of cardiac ultrasound indices in normal, overweight, and obese populations. (a) Aorta (AO). (b) Left Atrium (LA). (c) Right Atrium (RA). (d) Right Ventricle (RV). (e) End Diastolic Diameter of Left Ventricle (EDD). (f) Main Pulmonary Artery (MPA). ^*∗∗*^*P* < 0.001. ^*∗*^*P* < 0.05 ns *P* > 0.05.

**Figure 2:**
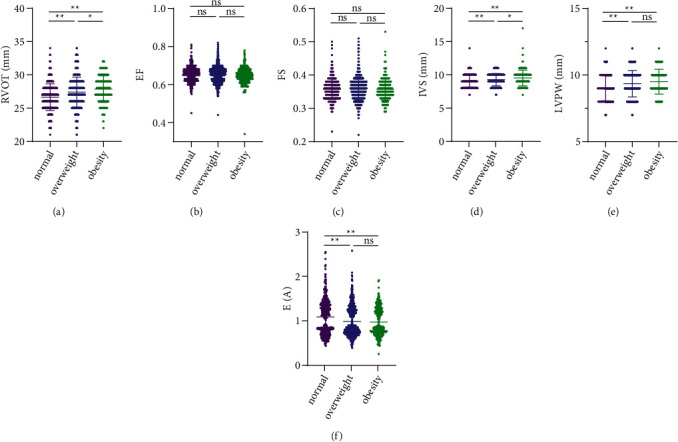
Comparison of cardiac ultrasound indices in normal, overweight, and obese populations. (a) Right ventricular outflow tract (RVOT). (b) Ejection fraction (EF). (c) Fractional shortening (FS). (d) Interventricular septum (IVS). (e) Left ventricular posterior wall (LVPW). (f) E/A Values. ^*∗∗*^*P* < 0.001. ^*∗*^*P* < 0.05 ns *P* > 0.05.

Being obese or overweight significantly increased AO, LA, RA, RV, EDD, MPA, RVOT, IVS, and LVPW, and decreased E/A values compared to participants with normal BMI (*P* < 0.001). Compared to overweight participants, obesity significantly increased LA and EDD (*P* < 0.001) and slightly increased RV, MPA, RVOT, and IVS (*P* < 0.05), while AO, RA, LVPW, and E/A values were not significantly different between the two groups (*P* > 0.05). EF and FS were not significantly different between the three groups (*P* > 0.05). All cardiac ultrasound indices for the three groups are shown in Figures 1 and 2.

**Figure 3:**
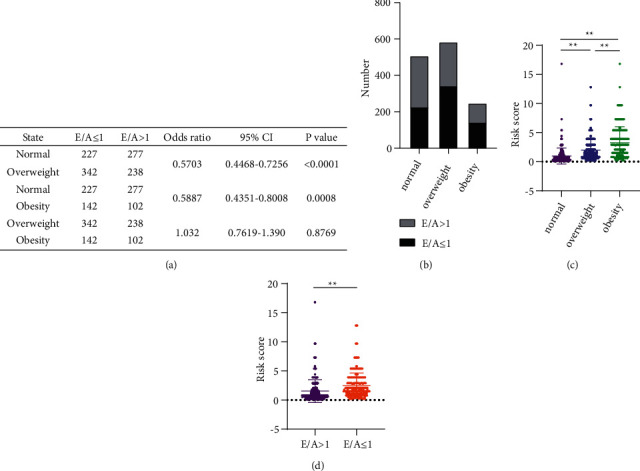
Cardiac ultrasound parameters and cardiovascular risk. (a) Results of chi-square tests for E/A ≤ 1 and E/A > 1 in normal, overweight, and obese populations. (b) Proportions of E/A ≤ 1 and E/A > 1 in normal, overweight, and obese populations. (c) Comparison of cardiovascular risk in normal, overweight and obese populations. (d) Comparison of cardiovascular risk for E/A ≤ 1 and E/A > 1. ^*∗∗*^*P* < 0.001.

Because E/A ≤ 1 was considered an early cardiac impairment, we compared the three groups based on E/A values. The chi-square test showed that obesity and overweight significantly reduced the E/A value group compared to normal individuals (*P* < 0.001), while there was no significant difference in E/A values between these two groups (*P* = 0.8769) (Figures 3(a) and 3(b)). Representative echocardiographic images are shown in Supplemental Figure 2.

### 3.3. Blood Indicators

**Figure 4:**
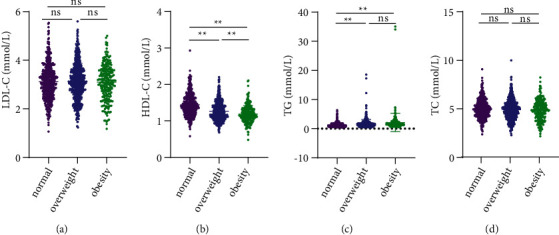
Comparison of lipid indices in normal, overweight, and obese populations. (a) Low-density lipoprotein-cholesterol (LDL-C). (b) High-density lipoprotein-cholesterol (HDL-C). (c) Triglycerides (TG). (d) Total cholesterol (TC). ^*∗∗*^*P* < 0.001 ns *P* > 0.05.

Obese and overweight people had higher TG and lower HDL-C levels (*P* < 0.001). Obesity and being overweight did not seem to affect LDL-C and TC in the healthy population (*P* > 0.05).  Figure 4 displays the blood lipid levels in the three groups of participants.

### 3.4. Baseline Characteristics of Participants for Cardiovascular Risk Assessment

Because the SCORE score can only assess cardiovascular risk in people aged 35–60 years, we screened eligible people from the included population. A total of 781 participants met the criteria, of which 301 were normal weight, 327 were overweight, and 153 were obese. All participants' age, gender, BMI, systolic blood pressure, TC, tobacco use, cardiac ultrasound parameters, and risk scores are presented in Table 2.

### 3.5. Cardiac Ultrasound Parameters and Cardiovascular Risk

**Figure 5:**
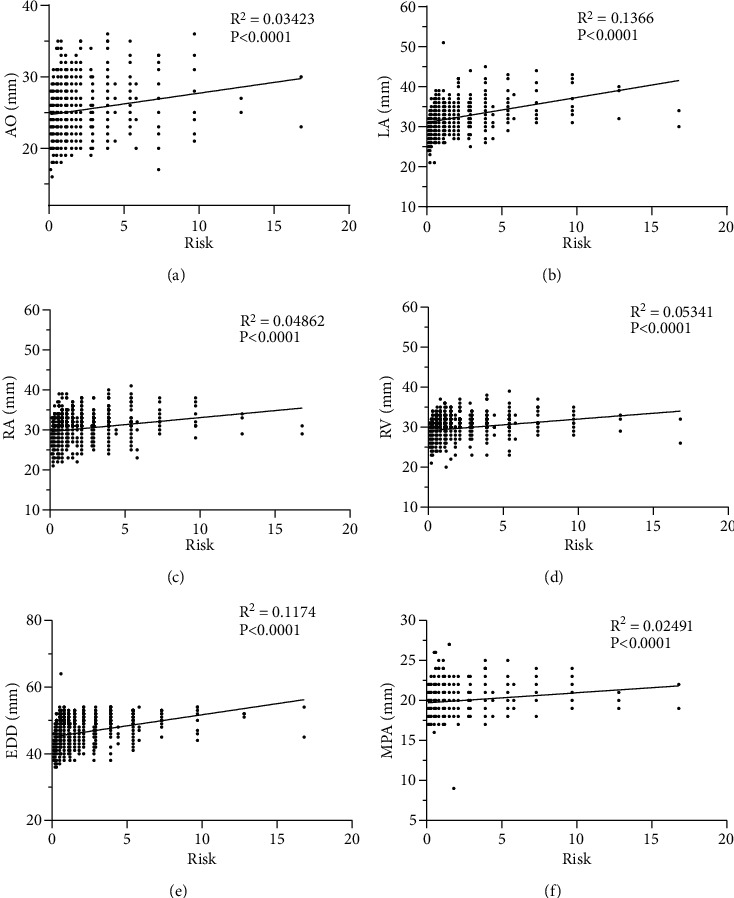
Correlation of cardiac ultrasound parameters with cardiovascular risk. (a) Aorta (AO). (b) Left atrium (LA). (c) Right atrium (RA). (d) Right ventricle (RV). (e) End diastolic diameter of left ventricle (EDD). (f) Main pulmonary artery (MPA).

**Figure 6:**
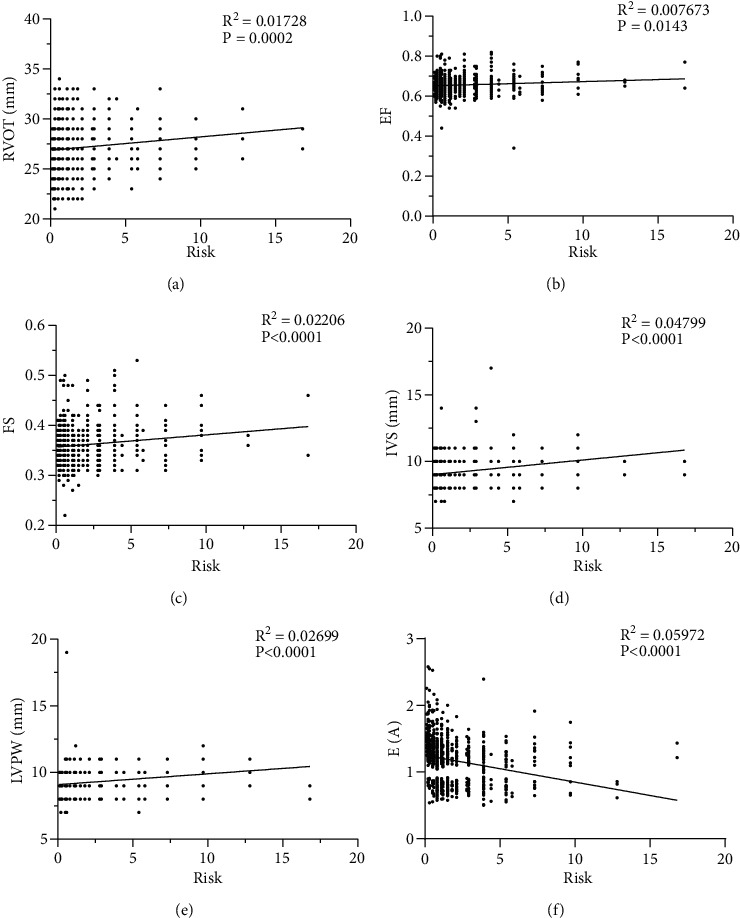
Correlation of cardiac ultrasound parameters with cardiovascular risk. (a) Right Ventricular Outflow Tract (RVOT). (b) Ejection Fraction (EF). (c) Fractional shortening (FS). (d) Interventricular septum (IVS). (e) Left ventricular posterior wall (LVPW). (f) E/A values.

Cardiovascular risk increased significantly with increasing body weight and was significantly higher for E/A ≤ 1 than for E/A > 1 (*P* < 0.001) (Figures 3(c) and 3(d)). The results of the correlation analysis showed that AO, LA, RA, RV, EDD, MPA, RVOT, FS, IVS, and LVPW were significantly associated with cardiovascular risk, which increased significantly with the increase of these indicators (*P* < 0.001). The association between EF and cardiovascular risk was relatively weak (*P*=0.0143). E/A values and cardiovascular risk showed a significant negative correlation (*P* < 0.001). Figures 5 and 6 show scatter plots of the correlation between cardiac ultrasound indices and cardiovascular risk.

## 4. Discussion

Obesity is a major health problem that requires immediate care because it is a noncommunicable condition that is currently quite common and can cause a number of cardiovascular problems. It affects a person's appearance and quality of life as well. Obese individuals have higher rates of diabetes, dyslipidemia, and cardiovascular diseases than individuals of normal weight [9]. An imbalanced diet, bad eating habits, and insufficient physical activity are the main causes of obesity. By accumulating a lot of fat around the heart, pericardium, and main vessels, long-term obesity, especially abdominal obesity, causes the heart to expand, increase load and resistance, affect the compliance of the heart muscle, and ultimately lead to structural changes [4]. Additionally, eating too many foods high in fat and cholesterol raises blood lipid levels, and hyperlipidemia can result in endothelial cell injury and localized detachment, lipid plaque formation, and smooth muscle cell proliferation, all of which combine to generate atheromatous plaques [10]. In this study, we discovered that whereas LDL-C and TC levels did not substantially differ across the three groups, obesity and being overweight raised TG levels and lowered HDL-C levels. Inflammatory response chemicals have been found to increase with weight in the blood and tissues of obese individuals. These substances can affect the insulin receptor's sensitivity and conduction mechanism, leading to insulin resistance, which is recognized to be one of the causes of metabolic syndrome and later makes type 2 diabetes more likely [11]. By interfering with the heart's ability to repolarize, elevated free fatty acids can also result in arrhythmias and the death of cardiomyocytes [12]. Increased oxidative stress, lipids, and inflammation all contribute to increased synthesis of lipotoxic compounds, myocardial and signaling system damage, heart failure, and arrhythmias. Due to the increased risk of arrhythmias caused by sympathetic and parasympathetic dysfunction, obese people are much more likely to die suddenly [13]. The fact that overweight and obesity account for 14% of heart failure in women and 11% of heart failure in men when other cardiovascular factors are taken into consideration is proof that obesity is a cause of heart failure [14].

Changes in the heart's structure and function brought on by obesity frequently occur well before clinical symptoms. Our study showed that obesity and overweight can significantly alter the anatomy and function of the heart in a healthy population. The enlargement of the LA and RA may be associated with a higher risk of atrial fibrillation in the future [15]. The increase in AO, EDD, MPA, RVOT, IVS, and LVPW may be related to compensatory modifications brought on by the higher cardiac burden associated with obesity, and the increase in these indices is also closely related to the future emergence of structural heart disease [16]. While EF and FS have not yet experienced a significant decrease, the decline in E/A values suggests a decline in early cardiac diastolic function, suggesting that the diastolic function of the heart is primarily impacted in the early stages of obesity or being overweight [17]. If prompt action is not taken, the loss of heart function at this stage could lead to irreversible cardiac damage. The structural and functional signs of the heart can be measured with cardiac ultrasonography in a quick and precise manner, so they can be periodically checked. A sophisticated imaging technique called speckle tracking echocardiography (STE) can identify changes in cardiac deformation during the subclinical phase. Compared to two-dimensional conventional echocardiography, STE is regarded as a validated approach for assessing the left atrium and left ventricle [18–20]. However, STE is not yet universally available for the physical examination population, but in the future, it will be more useful as a routine screening method for the assessment of cardiac structure and function. Therefore, this study used cardiac 2D echocardiography to screen for cardiac structure and function in a healthy population. This study shows that the heart structure and function tend to decrease with increasing body weight, but the heart is still in the compensatory stage, and early intervention can prevent irreversible damage to the heart structure and function. As a result, heart structure and function may already be impaired in obese or overweight people, even if there may be a considerable time before any visible clinical signs appear. Early cardiac ultrasound testing can determine whether there are any early signs of heart damage in this population, allowing for more precise therapies to lower the risk of developing future cardiovascular problems.

Based on the patient's clinical data, including sex, age, smoking status, systolic blood pressure, and TC, the SCORE system calculates the likelihood that a fatal cardiovascular event will occur within the next 10 years [21, 22]. We further selected and included participants and evaluated cardiovascular risk because the SCORE method can only perform risk assessment in people aged 35–60. Our findings demonstrated that participants with E/A ≤ 1 had significantly higher cardiovascular risk than participants with E/A > 1, which further shows that an early deterioration in diastolic function may be a risk factor for the later onset of cardiovascular disease. The positive and significant relationships between AO, LA, RA, RV, EDD, MPA, RVOT, IVS, and LVPW and cardiovascular risk imply that early anomalies in cardiac structure can raise the likelihood of future cardiovascular events. We postulate that cardiac structural and functional impairment brought on by weight gain in healthy people is positively linked with future cardiovascular disease risk. Obesity or being overweight is associated with early cardiac structural and functional impairment.

This study also has some shortcomings. First, since this is a cross-sectional study, causality cannot be established. The SCORE system, secondarily, only provides an estimate of cardiovascular risk and may not accurately reflect the incidence of upcoming cardiovascular events. A bigger sample size and numerous indicator monitoring will be required in the future to validate the outcomes of this study because all participants were only monitored for indicators once and the sample size was limited. Finally, advanced imaging modalities such as 3D-STE and cardiac MRI were not used in the article.

In conclusion, being obese or overweight is associated with early damage to cardiac structure and function and an increased risk of developing cardiovascular disease over the next 10 years. Early cardiac ultrasound testing can therefore help obese or overweight individuals better understand the structure and function of the heart and offer appropriate interventions to lower the risk of cardiovascular events.

## 5. Conclusion

In populations that are otherwise healthy, being obese or overweight can result in early heart structural and functional deterioration and raise the likelihood of future cardiovascular events. Cardiovascular events in obese or overweight populations can be prevented and treated using the substantial link between impaired heart shape and function and events as shown by cardiac ultrasonography as a guiding parameter.

## Figures and Tables

**Table 1:** Comparison of baseline characteristics and cardiac ultrasound indices in a stratified population based on BMI.

	Normal (*n* = 504)	Overweight (*n* = 580)	Obesity (*n* = 244)
Demographics			
Age (y)	56.57 (13.98)	57.72 (12.49)	55.58 (12.51)
Male	266 (52.78%)	442 (76.21)	206 (84.43%)
BMI (kg/m^2^)	21.85 (1.66)	25.78 (1.08)	30.1 (2.04)
SBP (mmHg)	121.88 (17.18)	127.91 (16.2)	130.45 (17.23)
TC (mmol/L)	5.02 (0.97)	4.94 (1.02)	4.86 (1.03)
TG (mmol/L)	1.28 (0.77)	1.73 (1.45)	2.11 (3.15)
LDL-C (mmol/L)	3.12 (0.72)	3.13 (0.74)	3.1 (0.73)
HDL-C (mmol/L)	1.45 (0.3)	1.26 (0.26)	1.19 (0.03)
Smoking (%)	87 (17.26%)	147 (28.94%)	89 (36.48%)

Echocardiography			
AO (mm)	24.63 (3.22)	25.68 (3.47)	26.11 (3.5)
LA (mm)	31.24 (3.85)	33.19 (3.36)	35.07 (3.72)
RA (mm)	29.16 (3.2)	30.86 (3.38)	31.14 (3.43)
RV (mm)	28.76 (2.51)	30.14 (2.45)	30.68 (2.8)
EDD (mm)	44.83 (3.89)	47.37 (3.69)	48.4 (3.53)
MPA (mm)	19.67 (1.52)	20.11 (1.74)	20.59 (2.6)
RVOT (mm)	26.63 (2.01)	27.4 (2.26)	27.86 (2)
EF	0.65 (0.04)	0.65 (0.04)	0.65 (0.44)
FS	0.36 (0.03)	0.36 (0.04)	0.36 (0.03)
IVS (mm)	9.05 (0.97)	9.29 (0.93)	9.54 (1.18)
LVPW (mm)	9.03 (1.04)	9.5 (0.93)	9.5 (0.93)
E/A	1.09 (0.39)	0.99 (0.34)	0.97 (0.32)

Abbreviations. BMI, body mass index; SBP, systolic blood pressure; TC, total cholesterol; TG, triglycerides; LDL-C, low-density lipoprotein-cholesterol; HDL-C, high-density lipoprotein-cholesterol; AO, aorta; LA, left atrium; RA, right atrium; RV, right ventricle; EDD, end diastolic diameter of left ventricle; MPA, main pulmonary artery; RVOT, right ventricular outflow tract; EF, ejection fraction; FS, fractional shortening; IVS, interventricular septum; LVPW, left ventricular posterior wall.

**Table 2:** Comparison of baseline characteristics and cardiac ultrasound parameters in the population undergoing SCORE system scoring.

	Normal (*n* = 301)	Overweight (*n* = 327)	Obesity (*n* = 153)
Demographics			
Age (y)	47.22 (7.29)	48.91 (6.82)	47.96 (6.98)
Male	138 (45.85%)	256 (78.29)	136 (88.89%)
BMI (kg/m^2^)	21.82 (1.63)	25.73 (1.08)	30.28 (2.08)
SBP (mmHg)	116.6 (14.9)	123.1 (14.46)	127.1 (14.88)
TC (mmol/L)	5.05 (0.92)	5.03 (1)	4.93 (0.96)
Smoking (%)	56 (18.16%)	92 (28.13)	63 (41.18%)

Echocardiography			
AO (mm)	24.32 (3.12)	25.74 (3.4)	26.07 (3.52)
LA (mm)	30.56 (3.1)	32.56 (3.09)	34.84 (3.36)
RA (mm)	28.95 (3.12)	30.98 (3.12)	31.12 (3.37)
RV (mm)	28.67 (2.56)	30.18 (2.31)	30.61 (2.7)
EDD (mm)	44.25 (3.62)	47.16 (3.8)	48.54 (3.48)
MPA (mm)	19.58 (1.48)	20.05 (1.76)	20.41 (1.65)
RVOT (mm)	26.54 (2.04)	27.29 (2.44)	27.74 (1.82)
EF	0.66 (0.04)	0.66 (0.06)	0.65 (0.04)
FS	0.36 (0.03)	0.36 (0.04)	0.36 (0.03)
IVS (mm)	9 (0.97)	9.24 (0.92)	9.61 (1.27)
LVPW (mm)	8.97 (1.08)	9.35 (0.96)	9.56 (0.92)
E/A	1.26 (0.35)	1.49 (6.57)	1.11 (0.3)
Risk	0.96 (1.37)	1.99 (1.86)	3.29 (2.74)

Abbreviations. BMI, body mass index; SBP, systolic blood pressure; TC, total cholesterol; AO, aorta; LA, left atrium; RA, right atrium; RV, right ventricle; EDD, end diastolic diameter of left ventricle; MPA, main pulmonary artery; RVOT, right ventricular outflow tract; EF, ejection fraction; FS, fractional shortening; IVS, interventricular septum; LVPW, left ventricular posterior wall.

## Data Availability

Data supporting the results of this study are available upon request from the corresponding author.

## Supplementary Materials

Supplementary Materials Supplemental File 1. Flow chart of the included subjects. Supplemental File 2. Representative echocardiographic images.

## References

[1] Heiss C., Spyridopoulos I., Haendeler J. (2018). Interventions to slow cardiovascular aging: dietary restriction, drugs and novel molecules. *Experimental Gerontology*.

[2] Kurth T., Rist P. M., Ridker P. M., Kotler G., Bubes V., Buring J. E. (2020). Association of migraine with aura and other risk factors with incident cardiovascular disease in women. *Journal of the American Medical Association*.

[3] Rosengren A. (2021). Obesity and cardiovascular health: the size of the problem. *European Heart Journal*.

[4] Genoni G., Menegon V., Monzani A. (2021). Healthy lifestyle intervention and weight loss improve cardiovascular dysfunction in children with obesity. *Nutrients*.

[5] Schinzari F., Tesauro M., Cardillo C. (2021). Vasodilator dysfunction in human obesity: established and emerging mechanisms. *Journal of Cardiovascular Pharmacology*.

[6] Pan X., Chen X., Ren Q. (2022). Single-cell transcriptome reveals effects of semaglutide on non-cardiomyocytes of obese mice. *Biochemical and Biophysical Research Communications*.

[7] Podolec M., Siniarski A., Pająk A. (2019). Association between carotid-femoral pulse wave velocity and overall cardiovascular risk score assessed by the SCORE system in urban polish population. *Kardiologia Polska*.

[8] Ivanovs R., Kivite A., Ziedonis D., Mintale I., Vrublevska J., Rancans E. (2018). Association of depression and anxiety with the 10-year risk of cardiovascular mortality in a primary care population of Latvia using the SCORE system. *Frontiers in Psychiatry*.

[9] Piché M. E., Tchernof A., Després J. P. (2020). Obesity phenotypes, diabetes, and cardiovascular diseases. *Circulation Research*.

[10] Valanti E. K., Dalakoura-Karagkouni K., Siasos G., Kardassis D., Eliopoulos A. G., Sanoudou D. (2021). Advances in biological therapies for dyslipidemias and atherosclerosis. *Metabolism*.

[11] Kawai T., Autieri M. V., Scalia R. (2021). Adipose tissue inflammation and metabolic dysfunction in obesity. *American Journal of Physiology–Cell Physiology*.

[12] Pellegrini C. N., Buzkova P., Lichtenstein A. H. (2021). Individual non-esterified fatty acids and incident atrial fibrillation late in life. *Heart*.

[13] Koliaki C., Liatis S., Kokkinos A. (2019). Obesity and cardiovascular disease: revisiting an old relationship. *Metabolism*.

[14] Carbone S., Lavie C. J., Elagizi A., Arena R., Ventura H. O. (2020). The impact of obesity in heart failure. *Heart Failure Clinics*.

[15] Seewöster T., Spampinato R. A., Sommer P. (2019). Left atrial size and total atrial emptying fraction in atrial fibrillation progression. *Heart Rhythm*.

[16] Shah R., Nucifora G., Perry R., Selvanayagam J. B. (2018). Noninvasive imaging in cardiac deposition diseases. *Journal of Magnetic Resonance Imaging*.

[17] Jung M. H., Ihm S. H., Lee D. H. (2021). Sex-specific associations of obesity with exercise capacity and diastolic function in Koreans. *Nutrition, Metabolism, and Cardiovascular Diseases*.

[18] Doğduş M., Kılıç S., Vuruşkan E. (2019). Evaluation of subclinical left ventricular dysfunction in overweight people with 3D speckle-tracking echocardiography. *The Anatolian Journal of Cardiology*.

[19] Dogdus M., Diker S., Yenercag M., Gurgun C. (2021). Evaluation of left atrial and ventricular myocardial functions by three-dimensional speckle tracking echocardiography in patients with euthyroid Hashimoto’s thyroiditis. *The International Journal of Cardiovascular Imaging*.

[20] Dogdus M., Yenercag M., Akhan O., Gok G. (2020). Assessment of left atrial mechanics and left ventricular functions using 3D speckle-tracking echocardiography in patients with inappropriate sinus tachycardia. *The International Journal of Cardiovascular Imaging*.

[21] Wierzbicka-Chmiel J., Mizia-Stec K., Haberka M., Chmiel A., Mizia M., Gasior Z. (2009). The relationship between cardiovascular risk estimated by use of SCORE system and intima media thickness and flow mediated dilatation in a low risk population. *Cardiology Journal*.

[22] Visseren F. L. J., Mach F., Smulders Y. M. (2021). ESC Guidelines on cardiovascular disease prevention in clinical practice. *European Heart Journal*.

