# Supplementary figures and images for: Correlation between Cardiac Ultrasound Index and Cardiovascular Risk in Healthy Obese and Overweight Populations
Source: Int J Clin Pract. 2022 Sep 21;2022:2235994. doi: 10.1155/2022/2235994 (PMC9519315; doi:10.1155/2022/2235994)

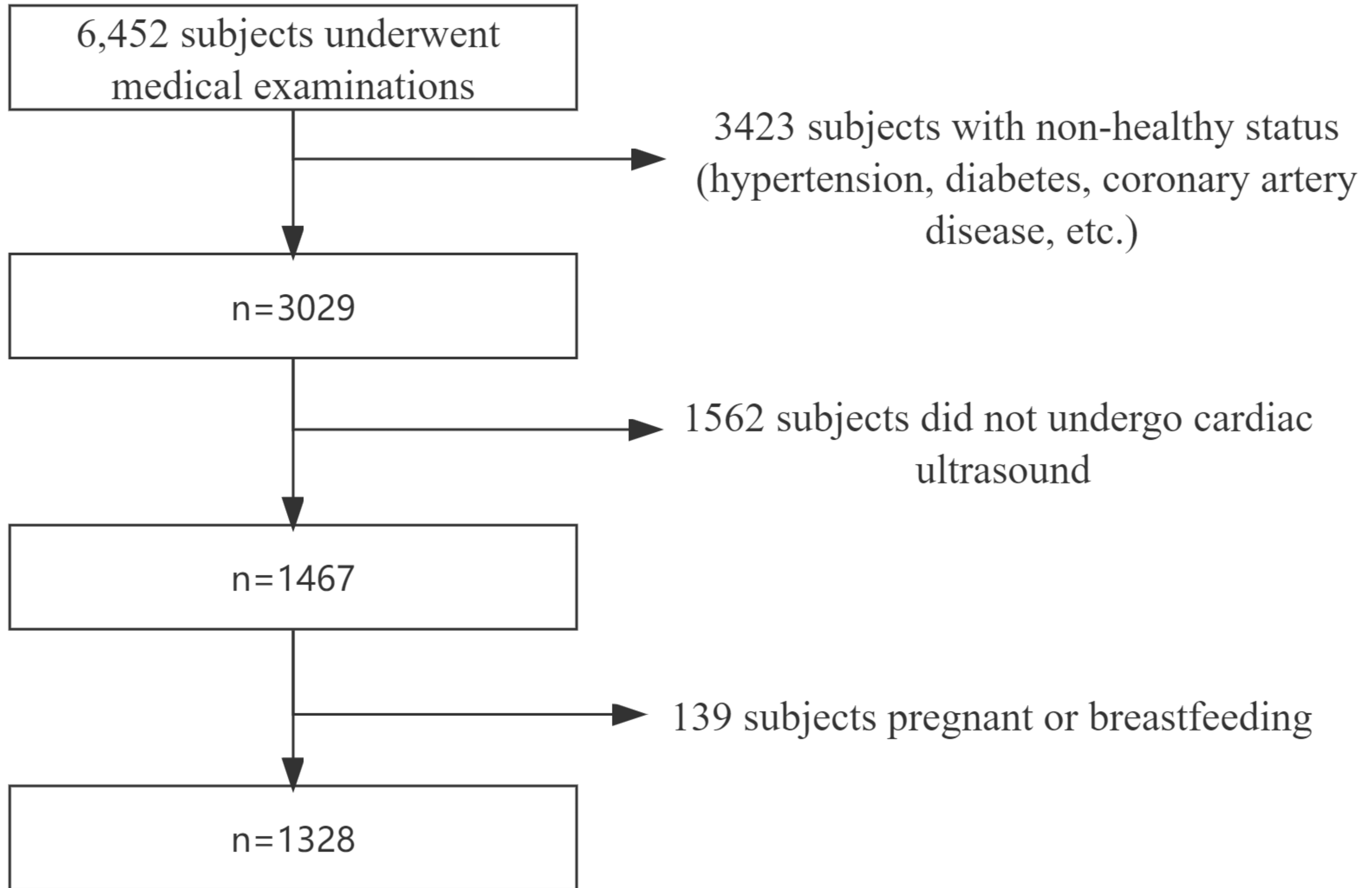

Supplement: Supplementary Materials — Supplemental File 1. Flow chart of the included subjects. Supplemental File 2. Representative echocardiographic images. [file 2235994.f1.zip › 2235994.f1/Supplemental Figure 1.pdf]

Normal

Overweight

Obesity

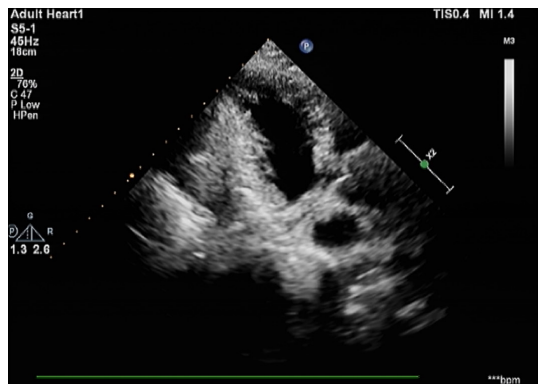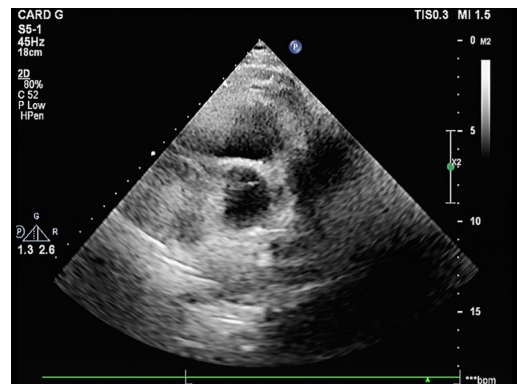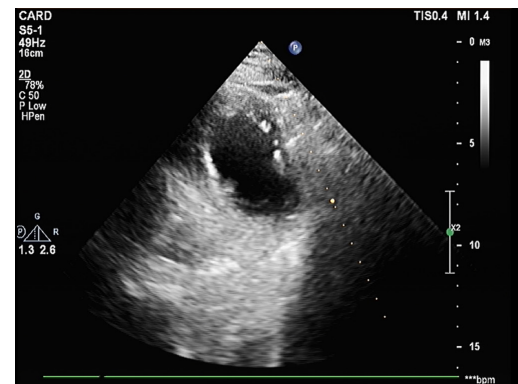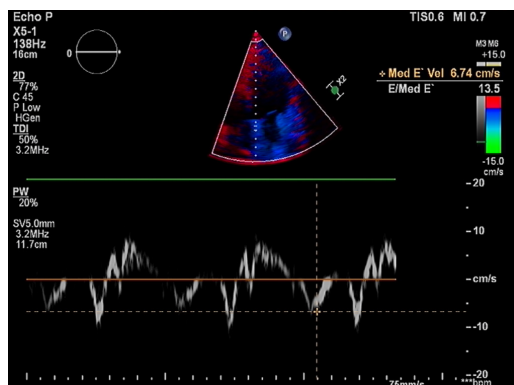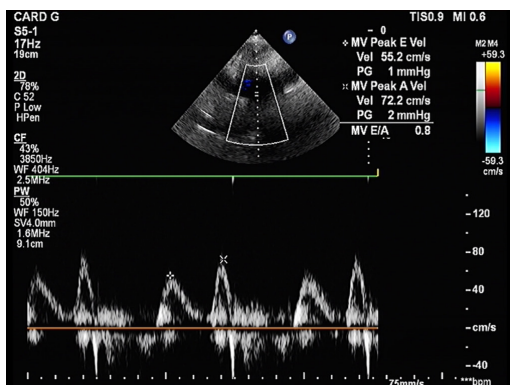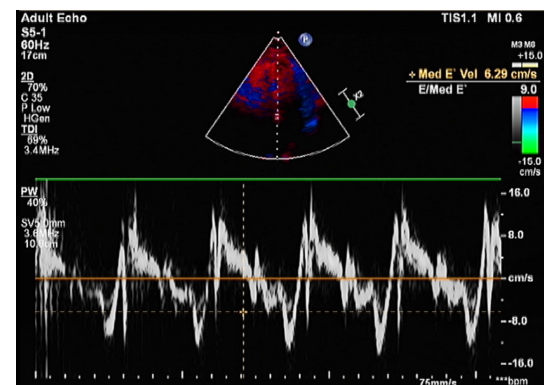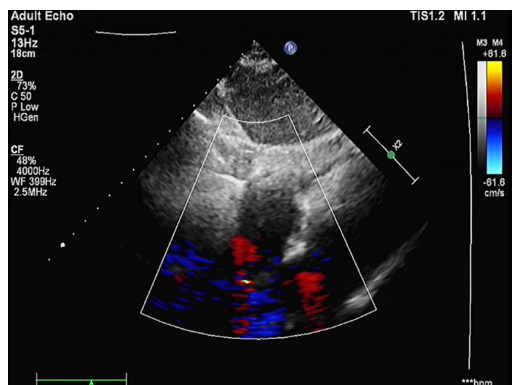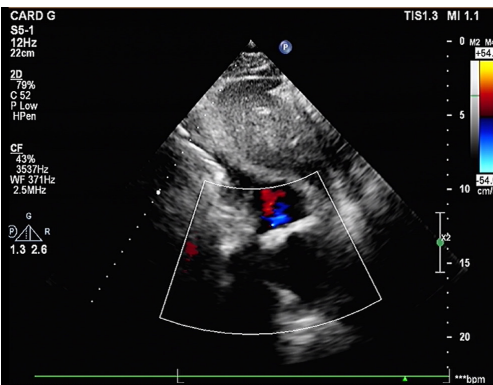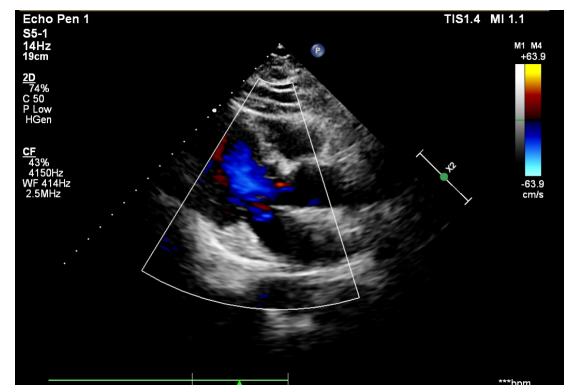

Supplement: Supplementary Materials — Supplemental File 1. Flow chart of the included subjects. Supplemental File 2. Representative echocardiographic images. [file 2235994.f1.zip › 2235994.f1/Supplemental Figure 2.pdf]
